# The Presence of Associated Injuries in Pediatric Radial Neck Fractures: A Systematic Review of the Literature and Meta-Analysis of Pooled Individual Patient Data

**DOI:** 10.3390/children12030300

**Published:** 2025-02-27

**Authors:** Lisette C. Langenberg, Joyce L. Benner, Nazira Bernal Bader, Christiaan J. A. van Bergen, Joost W. Colaris

**Affiliations:** 1 Department of Orthopaedics and Sports Medicine, Sophia Children’s Hospital, Erasmus Medical Center, dr. Molenwaterplein 40, 3015 GD Rotterdam, The Netherlands; 2 Centre for Orthopaedic Research Alkmaar (CORAL), Department of Orthopaedic Surgery, Northwest Clinics, Wilhelminalaan 12, 1815 JD Alkmaar, The Netherlands; 3 Department of Human Movement Sciences, Faculty of Behavioural and Movement Sciences, Vrije Universiteit Amsterdam, De Boelelaan 1105, 1081 HV Amsterdam, The Netherlands; 4 Department of Orthopedic Surgery, Clínica Alemana de Santiago de Chile, Universidad del Desarrollo, Av. Manquehue Nte 1410, Vitacura 7650567, Santiago de Chile, Chile; 5 Department of Orthopedic Surgery, Amphia, Molengracht 21, 4818 CK Breda, The Netherlands

**Keywords:** pediatric trauma, radial neck fracture, radial neck trauma, elbow injury

## Abstract

**Background:** Pediatric radial neck fractures (pRNFs) can occur in isolation or in association with concomitant injuries. It is unknown whether the presence of associated injuries should influence the choice of treatment. The aim of this study is to assess the incidence of associated injuries in pRNF and their correlation with fracture angulation (Judet grade) or the patient’s age (under or over ten years of age). **Methods:** A systematic literature review was performed following PRISMA-IPD guidelines, including case series on pRNF with a minimum of five cases of children until 16 years of age. The quality assessment included a risk of bias analysis and evaluation using the MINORS criteria. Individual patient data on age, Judet classification and associated injuries were extracted from the included studies and pooled for the meta-analysis. The correlation between the presence of associated injury and the patient’s age or Judet classification was depicted in two forest plots. **Results:** A total of 20 articles published sufficient individual patient data (n = 371) on associated injuries. All but one were retrospective case series. Fifteen articles had MINORS scores of 8 or higher. The incidence of associated injuries was 33% (123 of 371 cases). Almost half of the associated injuries included an olecranon fracture (61/123). There was no correlation between Judet classification (*p* = 0.243) and incidence nor between patient age and the incidence of associated injuries (*p* = 0.694). **Conclusions:** Surgeons should be aware of potential associated injuries in over a third of pRNF cases, regardless of the patient’s age or fracture angulation. Deduction of the trauma mechanism may be a more useful tool for assessing the potential presence of associated injuries than the most frequently used fracture classification or the patient’s age. More research is needed regarding the requirements for enhanced diagnostic imaging, specific treatment or follow-up adaptations in children with pRNFs and associated injuries.

## 1. Introduction

Radial neck fractures in children have been evaluated and treated for almost a century [1,2]. However, the criteria for the treatment choice remain a topic of debate. Numerous studies have reviewed the acceptable angulation of the radial neck and the related threshold for surgical intervention [3,4]. To date, it is still uncertain how the choice of treatment in pediatric radial neck fractures (pRNFs) should be altered if there are concomitant fractures or soft tissue injuries around the affected elbow. Case series and even reviews that have included associated injuries in their analyses report miscellaneous effects of associated injuries on the long-term outcome of pRNFs [4].

Because multiple ossification centers around the joint appear at different ages, diagnosing associated injuries in RNFs is even more complex in children [5,6]. In the literature, there is speculation that younger children are more prone to experiencing fractures as opposed to avulsion injuries due to the cartilaginous tissue in the physes [7]. Also, the type of avulsions or fractures that may be seen is susceptible to age [7,8,9]. Several authors report higher complication rates and unsatisfying outcomes in patients over ten years of age [10,11,12]. However, the impact of patient age on the incidence and type of associated injuries in pRNFs has never been studied.

In theory, a pRNF that is part of a more complex injury will require a different treatment from that for an isolated radial neck injury [11,13] because the presence of associated injuries may reflect a higher-energy trauma [11,14,15,16,17]. However, the most frequently used pRNF classifications are only based on radiologic criteria which do not include injuries to the surrounding ligaments or bones (Judet, [1], O’Brien [18] and AO Surgery [19,20], and some even include an overlap with those for intraarticular radial head lesions (Mason classification [21]). The current classification systems, such as the Judet grades, fail to address associated injuries, limiting their utility in guiding treatment. Even though it is presumed that higher fracture grades include a higher risk of associated injuries [4,22,23,24], there has never been an evaluation of the correlation between fracture angulation and the occurrence of associated injuries.

This study aims to provide a review of the literature and bridge this gap by analyzing the incidence and implications of associated injuries in pRNFs. The first aim is to evaluate whether there is a correlation between the incidence of associated injuries and patient age or Judet classification. Secondly, it aims to assess whether specific injuries are more prevalent in certain groups of patients based on Judet classification or age.

## 2. Materials and Methods

### 2.1. Systematic Search

**Figure 1:**
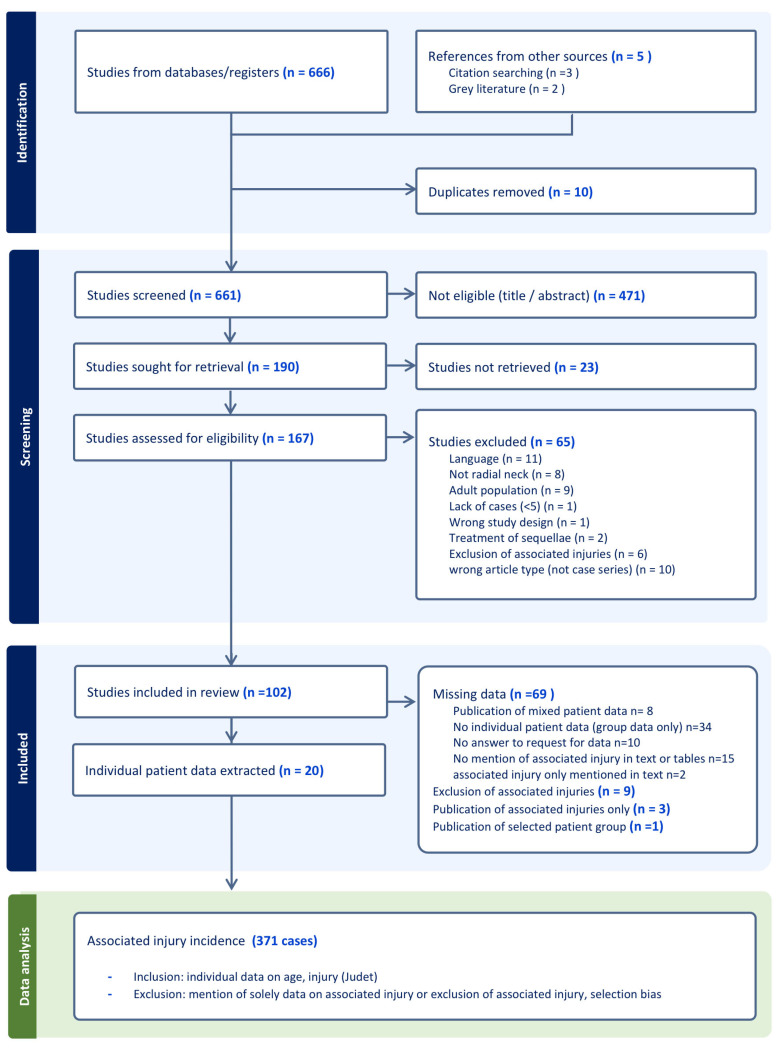
PRISMA-IPD guided search and specification of the inclusion of individual patient data for the analysis of the incidence of associated injuries (green rectangle). Mixed data: pRNF data published mixed with intraarticular fractures or data from adults (Mason classification was used).

This study followed the guidelines of the Preferred Reporting Items for Systematic reviews and Meta-analyses of Individual Patient Data (PRISMA-IPD [25]). Before starting the review process, the review was registered in PROSPERO (CRD42024572829). A systematic search was performed in Medline, Embase, Web of Science’s core collection, Cochrane Central Register of Controlled Trials and Google Scholar (Figure 1) in March 2024. Two separate reviewers (L.C.L. and N.B.B.) included case series with over 5 children that published sufficient data on the fracture (associated injuries, Judet classification) and patient age. The inclusion criteria were children < 16 years old with acute radial neck fractures. C.J.A.v.B. reviewed studies with a conflicting inclusion judgment as a third reviewer.

If solely group data were published without details on the individual patients, authors were contacted with a request to supply additional information via email. In cases without a response, these papers were excluded from the incidence analysis because no correction of the confounding factors was possible. Examples were as follows: selection bias in publications of non-consecutive cases, the publication of a select group of patients (only surgically treated cases, specific trauma mechanism or age group), etc.

### 2.2. Individual Patient Data Analysis: Associated Injury Incidence

Individual patient data on Judet grade and patient age were used for the meta-analysis. Three categories of initial fracture angulation were created based on the Judet grade: Judet 1 or 2 (fracture angulation of under 30 degrees), Judet 3 (fracture angulation of 30–60 degrees) and Judet 4 (fracture angulation of over 60 degrees).

Based on the previous literature that assumes a different injury mechanism over the age of ten [7,8,9], two age groups were created for children below and over the age of ten years to assess the influence of patient age on the occurrence of associated injuries.

### 2.3. Statistical Analysis

A statistical analysis was performed in R (Rstudio version 2024.04.2). An epidemiologic overview of the occurrence of associated injury was performed, identifying the most common lesions besides the radial neck fracture. A proportion meta-analysis explored the incidence of associated injuries with a random-effects model to determine whether subgroups of Judet classification or patient age showed different incidences. Forest plots were used to depict the correlation between the Judet classification or patient age and the presence of associated injury.

All of the tests were two-sided, and the significance of the statistical differences was attributed to a *p*-value of <0.05.

## 3. Results

### 3.1. Systematic Search

Following the title and abstract screening, 190 articles were sought for retrieval (Figure 1). Most of the studies that could not be retrieved had been published before 1980. A total of 102 case series were read and screened for eligibility for individual patient data extraction. Several of these case series solely presented group data (n = 34), which did not allow for an analysis of individual patient data. Thereby, ten case series were excluded because they did present some individual patient data but not all of the required inclusion criteria were met. The authors of these series were contacted by email with a request for the data. All ten were subsequently excluded because no reply was received. The IPD’s integrity was high; most of the case series published the same patient characteristics, even though some series dated before 2000. A quality assessment was performed using an adapted version of the MINORS criteria, mostly because all but one [26] of the case series were retrospective cohort studies (Table 1).

### 3.2. Analysis of the Incidence of Associated Injuries 

Several case series could not be used for the incidence analysis because inclusion bias applied. Nine authors mentioned the exclusion of multiple fractures or associated injuries [4,44,45,46,47,48,49,50,51]. Only one [52] indicated that the incidence of associated injuries was 34% amongst operated patients; two mentioned associated injuries in the text but did not indicate in which patients these occurred in the table of patient data [53,54]. Three case series contained associated injuries solely and could therefore not be included in the incidence analysis [11,55,56]. Fifteen articles did not mention whether associated injuries were present and were therefore excluded [57,58,59,60,61,62,63,64,65,66,67,68,69,70]. Eventually, 20 articles could be used for the analysis of the incidence of associated injuries in pRNFs (Table 1).

**Figure 2:**
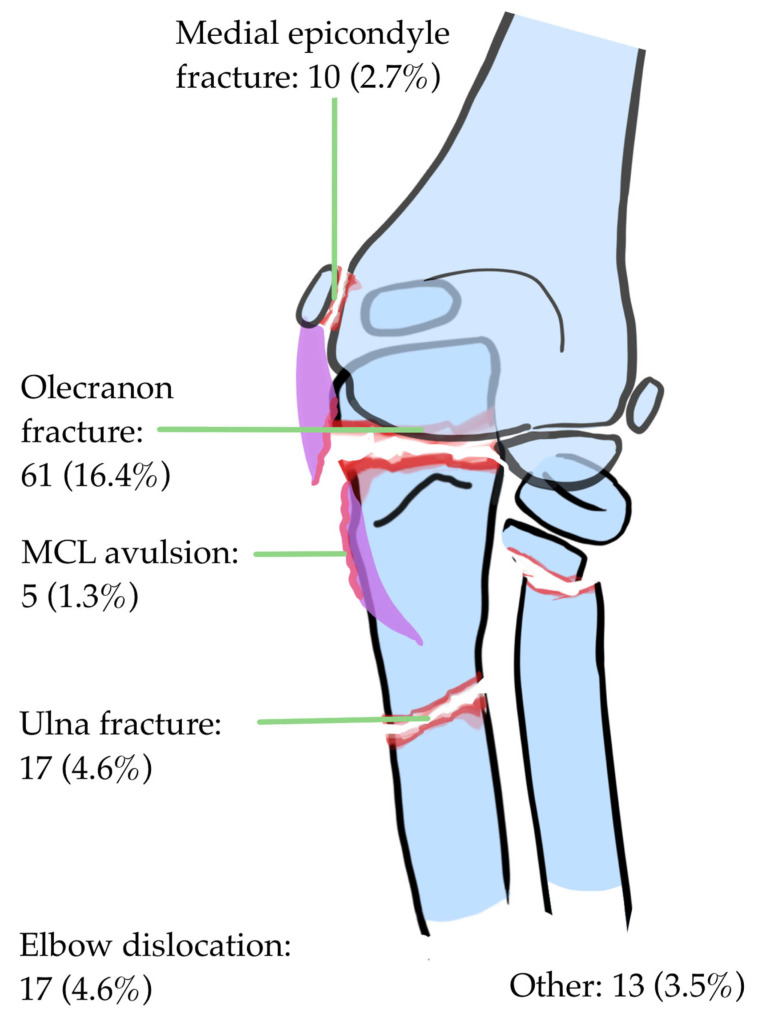
The incidence of associated injuries in pediatric radial neck fractures in a pooled analysis of 371 cases.

The incidence of associated injuries could be evaluated in individual patient data from 371 patients. Of these children, 123 had an associated injury (33.15%). There were 57 olecranon fractures, and an additional 4 olecranon fractures were part of an elbow dislocation. The second most frequent associated injury was an ulna fracture (other than olecranon; 14 cases; Table 2, Figure 2).

In both age groups, olecranon fractures were the most commonly associated injuries. Especially in very young children (<6 years, n = 37), olecranon fractures were diagnosed frequently (10/13 associated injuries in children < 7 years). Elbow dislocations were present more frequently in older children (3/205 in children under ten (1.5%) vs. 13/166 in children over ten (7.8%, CHI^2^ *p* = 0.003). The youngest child who had a dislocation was 7 years old.

**Figure 3:**
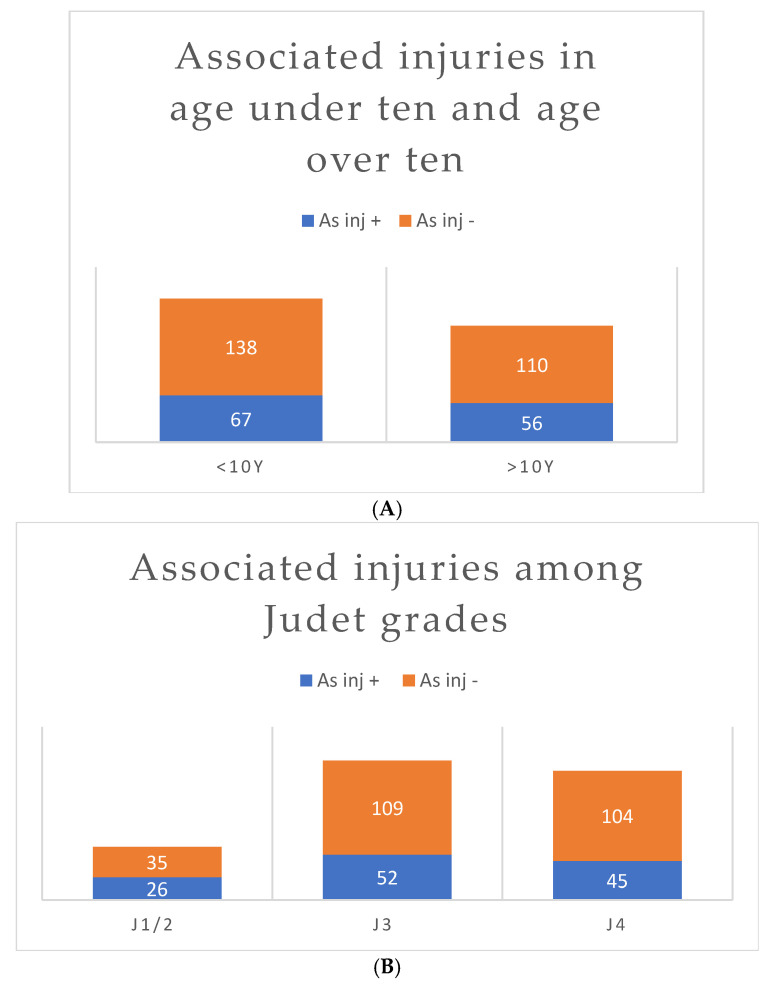
Distribution of cases among age categories and Judet grades (J). (**A**) A total of 67/205 patients under ten years of age had associated injuries, compared to 56/166 in patients over ten years of age (*p* = 0.694; forest plot is depicted in Figure 4A). (**B**) There is no significant difference between Judet grades for the incidence of associated injuries; *p* = 0.243; forest plot: Figure 4B.

**Figure 4:**
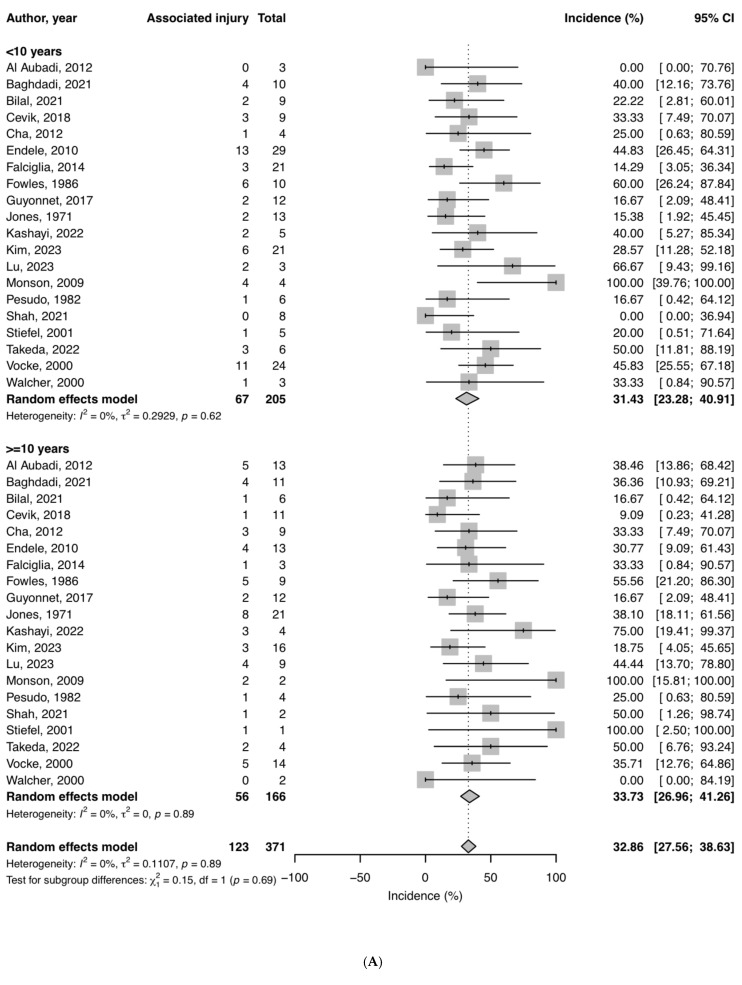

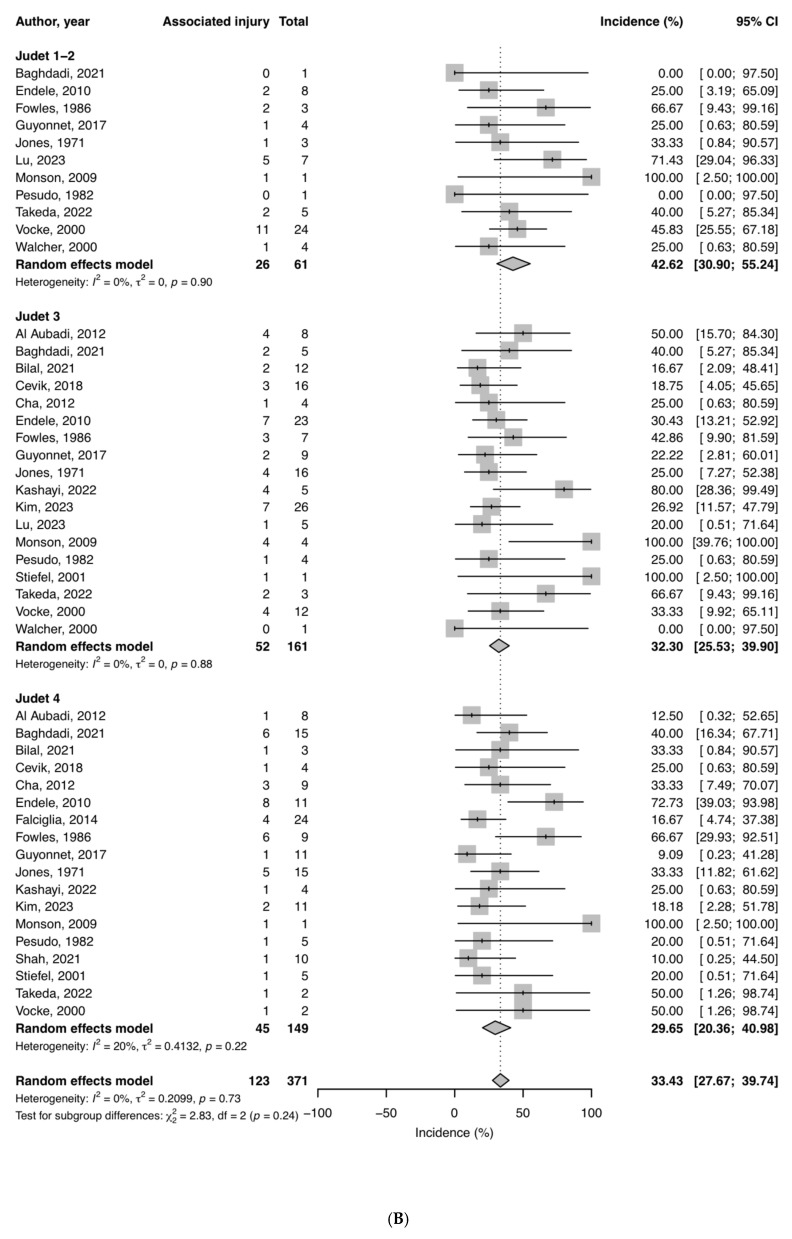
(**A**) Forest plot of meta-analysis of pooled individual patient data for patient age. There is no significant difference in the occurrence of associated injuries in patient age groups of under ten years or over ten years of age (*p* = 0.694). (**B**) Forest plot of meta-analysis of pooled individual patient data for fracture angulation. The Judet grade is not a predictor of the occurrence of associated injuries; CHI square *p* = 0.243 [10,22,26,27,28,29,30,31,32,33,34,35,36,37,38,39,40,41,42,43].

The incidence of associated injuries did not differ significantly between children under ten years compared to that in children older than ten years (Figure 3A and Figure 4A). The percentage of associated injuries was also not correlated with Judet grade (Figure 3B and Figure 4B).

## 4. Discussion

This is the first study that has used pooled individual patient data (IPD) to perform an epidemiological overview of the types of associated injuries in pRNFs and their incidence while correlating their presence with Judet grade or age. The meta-analysis of the IPD from 371 children with a pRNF showed that around one-third of these children had associated injuries, regardless of their Judet grade or age. Despite the fact that it has been speculated in the literature that a higher fracture angulation represents a more complex injury [4,22,23,24], there was no significant difference in the incidence of associated injuries between Judet 1/2, Judet 3 and Judet 4 pRNFs.

The incidence of associated injuries of 33% in our pooled data analysis complies with a previous review that analyzed the treatment outcomes in six case series of pRNFs [4] and found associated injuries in 36%.

The most commonly associated injuries are olecranon fractures, both in children under and over ten years. Dislocations are more frequently seen in older children; in our database, the first elbow dislocation was diagnosed in a seven-year-old child. In the previous literature, olecranon fractures and elbow dislocations have also been the most frequently reported associated injuries [23,42,71,72,73,74,75,76]. Less frequently, an associated proximal ulnar fracture, an ulnar shaft fracture or fractures of the lateral condyle of the humerus have been described [52,71].

### 4.1. Fracture Classification and Trauma Mechanism

**Figure 5:**
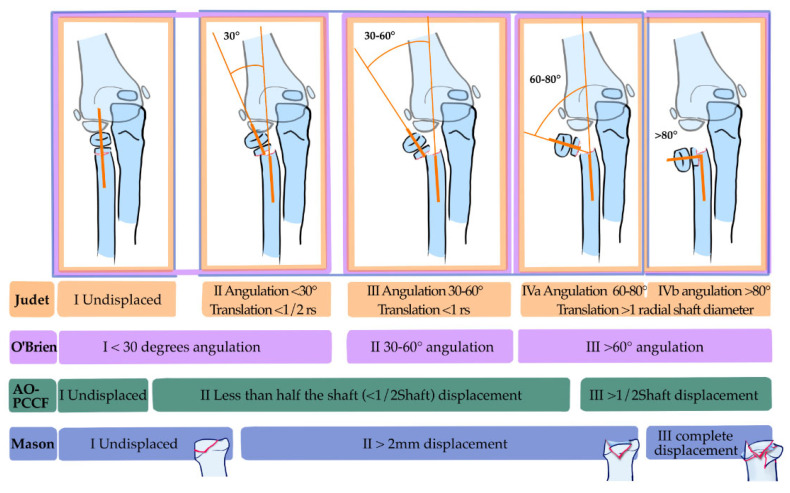
Overview of the most frequently used fracture classifications for pRNFs. Note that in the Mason classification, there is an overlap with intraarticular fractures of the radial head in the same classification. A Mason grade two fracture may be an intraarticular fracture with a >2 mm displacement or an extraarticular radial neck fracture with angulation. No angulation range for distinction between the three Mason grades has been specified. Following the AO-PCCF, a pRNF is coded as 21r: 2 (forearm), 1 (proximal) or r (Radius) and, depending on its location in the proximal radius, as E (epiphysis), M (metaphysis) or D (diaphysis). A separate code is added to specify the fracture pattern and the severity: I: undisplaced; II: a displacement of less than half the shaft diameter; III: a displacement of more than half the shaft diameter. rs = radial shaft. mm = millimeters.

The fact that the data analysis in this study shows that the Judet classification has a similar amount of associated injuries among each grade may be an argument for revising this classification system. The most widely used classifications for assessing fracture grade are Judet [1], O’Brien [18] and the AO Pediatric Comprehensive Classification of Long-Bone Fractures (AO-PCCF [77]). All are based on radiologic criteria obtained from plain X-rays (Figure 5). The Mason classification, meanwhile, is mainly a classification system for intraarticular fractures which include the radial head, but it may also be used to grade radial neck fractures. It lacks a specific range of angulation to distinguish between fracture grades: it simply defines “slightly angulated fractures” as grade two and severely angulated fractures as grade three [21,78]. There are multiple overlaps between these classification systems, which complicates an evaluation between the fracture grades of several classifications. For example, a grade 1 fracture according to the O’Brian classification may be a Judet grade 2 (Figure 5).

An important conclusion is that the current classifications fail to give insights into ligamentous injuries, which may lead to underestimation of the injury severity [8,11,79]. An example may be a pRNF following a reduced elbow dislocation: the X-rays will show a reduced joint without additional fractures. Many authors emphasize the importance of soft tissue to the functional outcome following a radial neck fracture [3,4,43]. For instance, interposition of the annular ligament has been described, which is not visible in radiographs [32]. An avulsion of the medial collateral ligament may become trapped in the ulnohumeral joint [8]. Using a classification system that does not address soft tissue injuries might result in missing these injuries at initial presentation [8]. The fact that the ossification centers around the elbow become visible in plain X-rays at varying ages may thereby further complicate an adequate diagnosis.

A classification system based on the trauma biomechanics may be more reliable in predicting which structures are potentially injured [80,81,82] and may aid in the detection of occult fractures [8]. In adults, deduction of the trauma mechanism and injury biomechanics has also been promoted to guide clinicians in optimizing the treatment [83,84]. In line with this, proposals have been made to also address ligamentous injuries in the Mason classification for radial head fractures [85,86].

**Figure 6:**
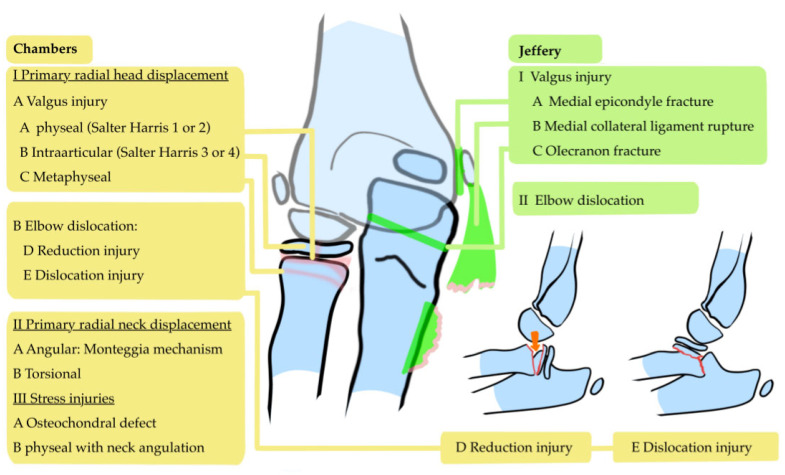
The Chambers classification and Jeffery classification. In these classifications, a valgus trauma mechanism is recognized with several associated injuries depending on the trauma mechanism.

Two classifications used less often include a deduction of the trauma mechanism. The Chambers classification distinguishes between a radial head displacement (a valgus injury or dislocation injuries) and a primary radial neck displacement. Still, it does not address the potential risk of associated injuries (Figure 6 [19,82,87]).

Jeffery’s classification notes the importance of medial collateral ligament injuries. An evaluation of two trauma mechanisms was published in 1972 [88], which identified compression and valgus load as the leading cause of pRNFs. Associated injuries in this classification included avulsion of the medial ligament (type 1A), which may include the medial epicondyle (type 1B) or an olecranon fracture (type 1C, Figure 6).

Both the Chambers and Jeffery’s classifications include a fracture type that occurs in elbow dislocation. A posterior shift or (sub)luxation of the elbow joint may cause the capitellum to catch the radial head and lead to a completely tilted radial neck [88]. An image of a laterally tilted radial head that is taken in full supination may mimic this fracture type. In these cases, there is a risk of aberrant fixation of the radial head in a reversed position, which is an essential pitfall of treatment [84].

**Figure 7:**
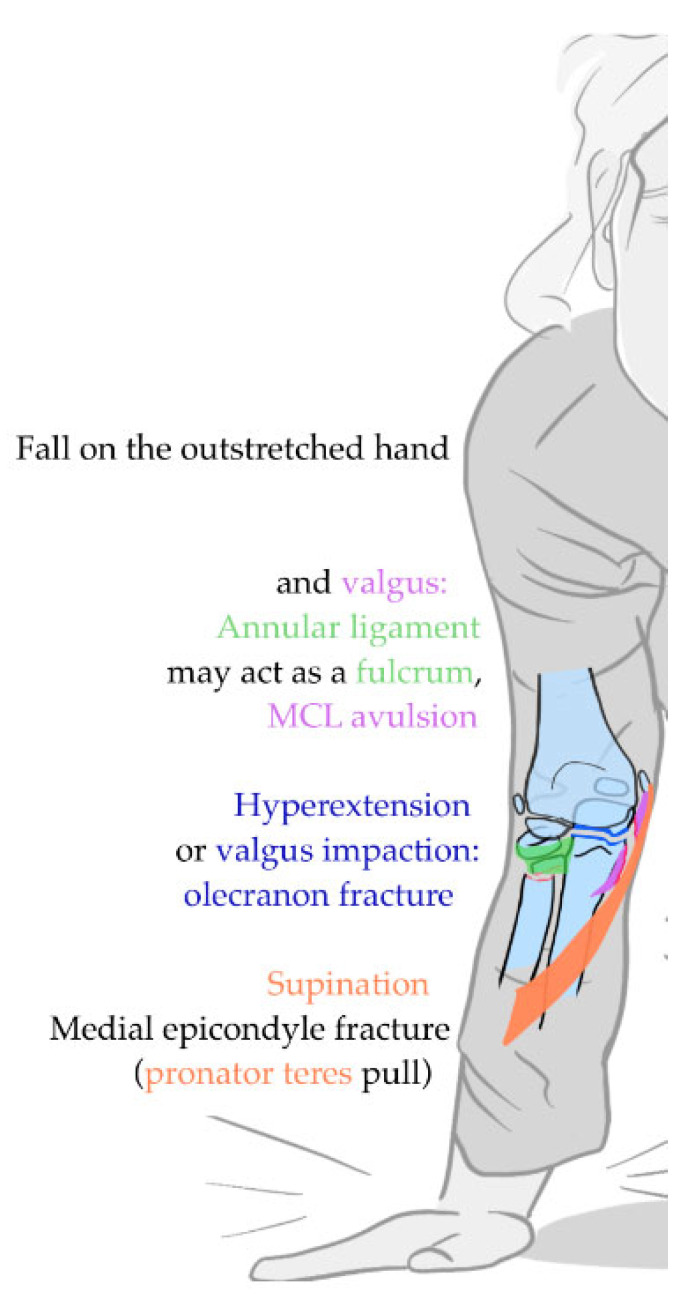
Most frequently described trauma mechanisms for pediatric radial neck fractures and explanation of potential biomechanic pathways of associated injuries. Note that the intact annular ligament may act as a fulcrum. An olecranon fracture may occur due to valgus compression or impingement on the olecranon fossa or as an avulsion of the triceps. Purple: medial collateral ligament (MCL); overlying orange muscle in valgus supination: pronator teres muscle; green shade overlying radial head: annular ligament.

It is generally agreed that pRNFs mainly occur due to valgus force [4,11,81,89], and the intact annular ligament may function as a fulcrum (Figure 7 [38,90]). It is presumed that the direction of pRNF angulation is determined by the rotation of the forearm during the fall [87]. In supination, the pull from the pronator teres muscles and flexor muscles may increase and hence cause avulsions or injury to the medial epicondyle [23,38,91].

Three additional “patterns of injury” have also been described, which include an anterior (sub)luxation of the elbow, a lateral shift of the lower arm or injuries including anterior radial head dislocation (Monteggia-like injuries [81]). The latter may be related to varus motion during trauma [8,79], whereas radial head fractures are mostly impaction injuries in hyperextension, often with concomitant fractures of the coronoid [85,86,92,93].

Monteggia injuries and Galleazi lesions should be seen as transverse unstable forearm injuries, which also include injury to the interosseous membrane [94]. In a Monteggia injury, there is a disruption of both the proximal radioulnar joint and the radiocapitellar joint [95], which should be seen as a different injury pathway. The treatment and follow-up of these lesions differ from the treatment of radial neck fractures, whose trauma mechanism is entirely different. While treating pRNFs, knowledge of the trauma biomechanics is therefore essential.

### 4.2. Types of Associated Injuries

Olecranon and ulna fractures occurred most frequently in the meta-analysis of this study, followed by elbow dislocations. Chambers stated that an olecranon fracture occurs due to compression [87], whereas hyperextension may also induce these types of fractures due to impingement on the olecranon fossa [79]. Olecranon fractures have also been reported in combination with elbow dislocations [4,96]. The fracture pattern of the olecranon may be important to the choice of treatment [95]. For example, shear fractures of the olecranon (Newman type 4 [95]) are unstable fractures that require internal fixation [81].

Soft tissue injuries are reported less frequently, potentially because these injuries are more frequently overlooked at initial presentation [8,79]. As noted, especially in very young children, diagnosing associated injuries may be difficult because history-taking may be more challenging; young children are generally more flexible, which complicates a diagnosis of instability; and a physical examination may be difficult in a child experiencing pain.

### 4.3. Associated Injuries and Influence on Outcome

The influence of associated injuries on the outcome following a pRNF is still debated. Studies that have evaluated the effect of associated injuries on the outcomes following a pRNF report variable outcomes. In a series that performed a univariate analysis, no significant difference in the outcome was described in eight studies [34,52,97,98,99,100,101,102], while a significantly worse outcome in pRNFs with associated injuries was found in two studies [33,100]. Others have provided descriptions of their outcomes without a statistical analysis; a poor outcome was reported in ten case series [10,14,17,22,43,62,103,104,105,106], and five found no influence [13,32,52,99].

One explanation for why an analysis of the outcome following a pRNF is complex is that there is a large confounding influence of treatment through open reduction (OR). Concomitant injuries may cause more pRNF instability and hence higher rates of ORs [71,107]. Thereby, the decision among surgeons to indicate an OR varies. Whereas most authors have described the presence of associated injuries as not influencing their choice of treatment [39], there have also been examples of surgeons who chose to perform an open reduction in every case with an associated injury [99,108]. In series where ORs could not have been an influencing factor, the results regarding the outcome of associated injuries are ambiguous. One case series that solely published data from patients that had an OR found no influence of the presence of associated injuries on their postoperative outcomes [99]. On the contrary, an evaluation of children who had a closed reduction showed that associated injury was still a predictor of a less favorable outcome [104]. It is thereby generally accepted that a higher complication rate is present following open reduction [12,15,16,100,106]. There is discussion on whether the worse results following ORs are caused by a more severe initial trauma or by the harmful effects of the surgical intervention [16,26,101]. Open surgery may cause damage to the vascularization or impairments to ROM due to the formation of fibrous tissue [36]. In a prospective study that included cases with 20 years of follow-up, functional impairment was mainly seen after growth disturbance following OR [26]. There may be an indication for intensified follow-up of this patient group because late sequalae of OR have been reported more often [14,24]. A multivariate analysis is needed that corrects for the influence of open surgery to clarify the influence of associated injuries on the outcomes following pRNFs.

Even though there is still discussion regarding the influence of associated injuries on the outcomes of pRNFs, the importance of diagnosing a ligamentous injury may be underestimated. Specific associated injuries may require an injury-specific treatment or follow-up. For instance, stiffness of the elbow may occur more often following an elbow dislocation [24]. It is important to realize that an elbow dislocation which involves a proximal radius fracture and a coronoid fracture (also known as the “unhappy triad”) is a rare injury in children and often results from a high-energy injury [76]. Thereby, if the medial collateral ligament is torn, the reduction in the displaced radial neck fracture may not be contained due to a lack of stability in the elbow [35,71]. In a study that evaluated the effect of ruptures of the medial collateral ligament (MCL), all pRNFs had MCL lesions in MRI scans that were performed at the moment of presentation [75]. Again, awareness of a potential ligamentous injury through deduction of the trauma mechanism may avoid missing these injuries.

There are some limitations to this study. Bias may apply because multiple case series on pRNFs that did not publish data on associated injuries could not be included. It is uncertain whether associated injuries were not present or whether they went unnoted. As explained above, many associated injuries may not have been diagnosed initially, and the incidence numbers may have been higher if MRI were used instead of only plain radiographs. However, in young children, it may be challenging to perform an MRI scan, especially in the acute setting. Thereby, mixed terminology and classifications that include intra- and extraarticular fractures [109], like the Mason classification [110], restricted the meta-analysis of the individual patient data. Heterogenous reporting [111], which is reflected in the low MINORS scores, complicated an analysis of patient and injury data other than those presented in this article. Some case series included Monteggia lesions [112], which were registered as associated injuries. It can be disputed whether the fracture classification was correct in these cases, which may have caused bias in the incidence calculations.

As pointed out before, associated injuries in radial neck fractures are frequently missed [8,11,17,75,80]. In one case series, no associated injuries were described, while one figure showed evident ulnar bowing [67]. Another pointed out that several associated medial epicondylar fractures were first diagnosed at follow-up after initially being missed [17]. It is possible that cases published as isolated radial neck injuries had, in fact, concomitant injury to the ligaments or epiphysis of the epicondyles that went unnoted. Future research focusing on the outcomes of pRNFs should include an accurate diagnosis of soft tissue injury at presentation using MRI. Ideally, prospective inclusion of consecutive patients with a set follow-up would provide clarity on the outcome of specific ligamentous injuries associated with radial neck fractures.

## 5. Conclusions

This study is the first to provide a meta-analysis of pooled individual patient data for associated injuries in pediatric radial neck injuries and assess the correlation between the incidence of associated injuries in pRNFs and the patient’s age or fracture angulation, as embedded into the Judet classification.

The incidence of associated injuries does not differ significantly in patients under and over ten years of age. Also, a higher Judet classification was not correlated with the presence of associated injuries, contrary to the currently accepted assumption that a higher fracture angulation may be related to a more complex injury.

This meta-analysis highlights the need for clinicians to adopt a trauma mechanism-based approach rather than relying solely on radiological classifications like the Judet classification. Not only will there be a smaller chance of missing associated injuries at presentation but the maltreatment of pRNFs associated with elbow dislocation may also be prevented. Future research should focus on integrating ligamentous and soft tissue injuries into comprehensive fracture classifications.

## Figures and Tables

**Table 1:** Overview of the included case series, including MINORS scores.

									MINORS							
Author	Year	Inclusion Range	Study Population	n	IPD Total	n AsInj	IPD AsInj	% AsInj	1	2	3	4: Outcome Scales	5	6	7	Total Score
Al Aubadi [27]	2012	2004–2008	displaced pRNF, treated with Metaizeau	16	16	5	5	31.25	1	1	2	Metaizeau, DASH, Steele	2	2	0	8
Baghdadi [28]	2021	2009–2018	pRNF that had OR	22	21	9	8	40.91	1	2	2	Flynn criteria, radiologic outcome	1	2	2	10
Bilal [29]	2021	2012–2014	pRNF > 30 gr, which could be managed using clamp leverage	15	15	3	3	20.00	1	1	1	Metaizeau radiologic criteria	2	0	2	7
Cevik [30]	2018	2007–2014	pRNF J3/4, CIMP + pKw leverage	20	20	4	4	20.00	1	2	2	Ursei, Tibone–Stoltz	2	0	2	9
Cha [31]	2012	2006–2008	pRNF treated with CIMP	13	13	4	4	30.77	2	1	2	Steele, ROM, radiologic criteria	2	0	0	7
Endele [32]	2010	1993–2006	pRNF treated with CIMP	54	42	21	17	38.89	1	1	2	Morrey and Metaizeau score	2	2	2	10
Falciglia [33]	2014	2000–2009	pRNF with OR after CR failed	24	24	4	4	16.67	1	1	1	ROM, own classification	2	0	2	7
Fowles [22]	1986	1965–1980	all pRNF	23	19	14	11	60.87	2	1	2	ROM, axis, radiological criteria	2	1	2	10
Guyonnet [34]	2017	2010–2015	pRNF treated with CIMP	24	24	4	4	16.67	1	2	2	Quick-DASH	1	0	2	8
Jones [35]	1971	1955–1969	displaced pRNF	34	34	10	10	29.41	1	1	2	ROM	2	2	2	10
Kashayi [36]	2022	2003–2009	pRNF treated with pKw leverage by one surgeon	9	9	5	5	55.56	1	2	2	ROM, MEPS	1	0	2	8
Kim [37]	2023	2012–2021	displaced pRNF	37	37	9	9	24.32	1	1	2	MEPS, Tibone–Stoltz	2	0	2	8
Lu [38]	2023	2015–2021	pRNF with medial epicondylar fracture	12	12	6	6	50.00	1	1	2	MEPS, ROM, carrying angle	2	0	2	8
Monson [39]	2009	nd	displaced pRNF	6	6	6	6	100.00	0	1	1	radiological RN reduction	0	0	0	2
Pesudo [40]	1982	nd	proximal radial epiphysis fractures	10	10	2	2	20.00	1	0	1	radiological criteria	2	0	2	6
Shah [41]	2021	2017–2018	completely displaced pRNF	10	10	1	1	10.00	2	1	1	ROM, radiologic union	2	0	0	6
Stiefel [10]	2001	1994–1996	J4 pRNF	6	6	2	2	33.33	2	1	2	own classification	1	0	0	6
Takeda [42]	2022	2005–2013	surgically treated pRNF	10	10	5	5	50.00	0	1	2	Leung/Peterson	2	0	2	7
Vocke [26]	2000	1984–1994	all pRNFs	38	38	16	16	42.11	1	1	1	ROM, complications	2	1	2	8
Walcher [43]	2000	1993–1996	displaced pRNF	5	5	1	1	20.00	1	1	1	G/P without further explanation, ROM	1	0	1	5
				388	371	224	123	33.15								

n: number of cases. IPD: individual patient data. Baghdadi, Fowles and Endele included cases with missing data, hence the difference in the total n and extracted IPD. As inj: associated injury. MINORS 1: Consecutive cases (1: patient selection process described in text; 2: consecutive cases). 2: Clear aim (1: aim stated but no clear outcome measures; 2: aim and outcome measures clearly stated). 3: Unbiased evaluation (0: subject to bias; 1: outcome measures may be subject to slight bias, like ROM or patient opinions on result; 2: clear outcome measures). 4: Outcome scales. 5: Follow-up published (1: group follow-up, 2: IPD of follow-up available). 6: Lost to follow-up (0: not mentioned; 1: mentioned without IPD of lost patients; 2: IPD of lost patients available).

**Table 2:** Incidence of associated injuries in 371 individual cases. MCL: medial collateral ligament.

Overview of the Incidence of Associated Injuries, n = 371			
Associated Injuries		Age < 10 y n = 205	**Age > 10 y** n = 166
**Olecranon fracture**	53	37	16
including wrist drop	1	1	
including lateral condyle fracture	2	1	1
including ipsilateral distal radius fracture	1		1
**Ulna fracture**	14	8	6
**Medial epicondyle fracture or avulsion**	8	1	7
Including lateral epicondyle fracture	1		1
**Lateral epicondyle fracture**	1		1
**Monteggia injury**	3	3	
**Radius and ulna fracture**	3	1	2
**Ipsilateral distal radius fracture**	2	1	1
**Coronoid fracture**	1		1
**Ipsilateral radial head**	1		1
**“Multiple”**	1		1
**Elbow dislocation**	16	3	13
including olecranon fracture	4	4	
including medial condyle fracture	1		1
including MCL tear	1		1
terrible triad	1	1	
**Ligament injuries**			
Ulnar collateral ligament injury	4	4	
Lateral collateral ligament injury	1		1
Cubitus valgus	1		1
**Neurologic injuries**			
Wrist drop	1	1	
Ulnar nerve injury	1	1	
**TOTAL**	**123**	**67**	**56**

## Data Availability

The database that was used for the individual patient data analysis is available for future research upon request.
